# Decipher identifies men with otherwise clinically favorable-intermediate risk disease who may not be good candidates for active surveillance

**DOI:** 10.1038/s41391-019-0167-9

**Published:** 2019-08-27

**Authors:** Annika Herlemann, Huei-Chung Huang, Ridwan Alam, Jeffery J. Tosoian, Hyung L. Kim, Eric A. Klein, Jeffry P. Simko, June M. Chan, Brian R. Lane, John W. Davis, Elai Davicioni, Felix Y. Feng, Peter McCue, Hyun Kim, Robert B. Den, Tarek A. Bismar, Peter R. Carroll, Matthew R. Cooperberg

**Affiliations:** 1 Department of Urology, University of California, San Francisco, Helen Diller Family Comprehensive Cancer Center, San Francisco, CA USA; 2 Department of Urology, Ludwig-Maximilians-University Munich, Munich, Germany; 3 GenomeDx Inc., Vancouver, BC Canada; 4 Department of Surgery, Johns Hopkins Medical Institutions, Baltimore, MD USA; 5 Department of Urology, University of Michigan, Ann Arbor, MI USA; 6 Department of Surgery, Cedars-Sinai Medical Center, Los Angeles, CA USA; 7 Glickman Urological and Kidney Institute, Cleveland Clinic, Cleveland, OH USA; 8 Department of Epidemiology and Biostatistics, University of California, San Francisco, Helen Diller Family Comprehensive Cancer Center, San Francisco, CA USA; 9 Urology, Spectrum Health Hospitals Prostate and Genitourinary Cancer Multispecialty Clinic, Grand Rapids, MI USA; 10 Department of Urology, Division of Surgery, The University of Texas MD Anderson Cancer Center, Houston, TX USA; 11 Department of Radiation Oncology, University of California, San Francisco, San Francisco, CA USA; 12 Department of Pathology, Anatomy and Cell, Thomas Jefferson University, Philadelphia, PA USA; 13 Department of Radiation Oncology, Thomas Jefferson University Hospital, Philadelphia, PA USA; 14 Department of Radiation Oncology, Washington University School of Medicine St. Louis, St. Louis, MO USA; 15 Departments of Pathology & Laboratory Medicine and Oncology, University of Calgary Cumming School of Medicine, Calgary, AB Canada

**Keywords:** Cancer genetics, Prognostic markers

## Abstract

**Background:**

We aimed to validate Decipher to predict adverse pathology (AP) at radical prostatectomy (RP) in men with National Comprehensive Cancer Network (NCCN) favorable-intermediate risk (F-IR) prostate cancer (PCa), and to better select F-IR candidates for active surveillance (AS).

**Methods:**

In all, 647 patients diagnosed with NCCN very low/low risk (VL/LR) or F-IR prostate cancer were identified from a multi-institutional PCa biopsy database; all underwent RP with complete postoperative clinicopathological information and Decipher genomic risk scores. The performance of all risk assessment tools was evaluated using logistic regression model for the endpoint of AP, defined as grade group 3−5, pT3b or higher, or lymph node invasion.

**Results:**

The median age was 61 years (interquartile range 56–66) for 220 patients with NCCN F-IR disease, 53% classified as low-risk by Cancer of the Prostate Risk Assessment (CAPRA 0−2) and 47% as intermediate-risk (CAPRA 3−5). Decipher classified 79%, 13% and 8% of men as low-, intermediate- and high-risk with 13%, 10%, and 41% rate of AP, respectively. Decipher was an independent predictor of AP with an odds ratio of 1.34 per 0.1 unit increased (*p* value = 0.002) and remained significant when adjusting by CAPRA. Notably, F-IR with Decipher low or intermediate score did not associate with significantly higher odds of AP compared to VL/LR.

**Conclusions:**

NCCN risk groups, including F-IR, are highly heterogeneous and should be replaced with multivariable risk-stratification. In particular, incorporating Decipher may be useful for safely expanding the use of AS in this patient population.

## Introduction

Men diagnosed with what has classically been termed “intermediate-risk” prostate cancer (PCa)—based on Gleason score 7, prostate-specific antigen (PSA) 10−20 ng/mL, and clinical stage T2b or 2c disease—have highly variable clinical behavior and prognosis and are considered a broad, heterogeneous cohort for whom management recommendations cannot be standardized. The literature provides strong evidence that not all Gleason sums of 7 have equal potential for progression. Men with a post radical prostatectomy (RP) Gleason 4 + 3 are more likely to develop metastasis and die from PCa than patients with a Gleason 3 + 4 [1], and outcomes may vary further based on the quantified predominance of pattern 4 disease [2, 3]. As a consequence, contemporary Gleason grading has explicitly assigned score 4 + 3 to a higher grade group (grade group 3, GG3) than Gleason 3 + 4 (GG2) to address these levels of risk [4]. Clinical T staging, meanwhile, has been shown frequently inaccurate, and less important than better markers of tumor volume, such as extent of biopsy core involvement [5].

The National Comprehensive Cancer Network (NCCN) now separates men with intermediate-risk PCa into favorable and unfavorable subgroups in their guidelines [6, 7]. Favorable-intermediate risk (F-IR) tumors are defined as GG1 or 2 tumors and no more than one NCCN intermediate-risk factor (PSA 10−20 ng/mL, cT2b/c, or GG2) and percentage of positive biopsy cores <50%. The latter parameter was an arbitrary addition that makes clinical sense but has never been validated to be an optimal threshold, and in fact no other NCCN stratum uses percent of cores involved. Studies have shown that men with F-IR PCa have similar survival outcomes compared to low-risk (LR) PCa patients, suggesting that some of these men may be appropriate candidates for active surveillance (AS) [8]. Conversely, patients with unfavorable intermediate-risk features demonstrate prostate cancer-specific and all-cause mortality rates similar to men with high-risk PCa [9]. Unsurprisingly, recent study groups have demonstrated that NCCN F-IR tumors have significantly worse odds of adverse pathology (AP) as compared to NCCN very low (VL) and LR tumors and these men had a small but measurable decrease in overall survival [10, 11]. Therefore, the suitability of men with F-IR tumors for AS remains controversial.

Genomic classifiers (GC) were introduced into clinical practice to improve risk-stratification and to help guide treatment decisions for men with PCa. Among them, Decipher, which uses a whole-transcriptome microarray assay, represents the most intensively studied GC [12]. It generates a score ranging from 0 to 1, with higher values indicating an increased probability for both AP and downstream oncologic outcomes. Utilization of molecular profiling with Decipher GC can result in improved identification of patients qualifying for AS by identifying the subset of histologically LR PCa at diagnosis with molecular characteristics confirming indolent disease [13].

In this study, we aimed to validate the Decipher GC to predict adverse pathology after RP in men with NCCN F-IR prostate cancer. Further, we investigated whether combining Decipher with a multivariable clinical risk-stratification tool can substratify AP risk, identifying which men with F-IR tumors may safely undergo AS.

## Materials and methods

### Study cohort

A multi-institutional PCa biopsy dataset was selected from the Genomic Resource Information Database (GRID, NCT02609269) consisting of 647 patients diagnosed with NCCN VL/LR or F-IR risk from 1990 to 2016 whose biopsies have undergone the Decipher testing and who received RP as first treatment. NCCN VL/LR was defined as follows: cT2a or lower, GG1, and PSA <10 ng/mL; NCCN F-IR was defined as GG1 or 2 and no more than one of the NCCN intermediate-risk factor (PSA 10−20 ng/mL, cT2b/c or GG2) and <50% biopsy cores positive. Pathologic endpoints were abstracted from surgical pathology reports. The research protocol was approved by institutional review boards of the participating institutions (University of California, San Francisco; University of Calgary; Johns Hopkins University; Cedars-Sinai; Cleveland Clinic; Spectrum Health; Thomas Jefferson University; MD Anderson Cancer Center).

Specimen collection and sample processing were conducted as described previously [13, 14]. The prostate needle biopsy core with highest grade and percentage of core involved with tumor was sampled for the Decipher assay, a CAP/CLIA clinical-grade whole-transcriptome assay. GC scores were generated based on a previously locked and validated signature (GenomeDx, San Diego, CA, USA), for all specimens [15].

### Statistical analysis

The primary objective was to evaluate Decipher’s prognostic ability to predict AP (defined as GG 3−5, pT3b or higher, or lymph node invasion (LNI) [14]) at RP within the NCCN F-IR group while accounting for clinical risk using the linear, extensively validated Cancer of the Prostate Risk Assessment (CAPRA [16]) score. In addition to this definition of AP, we also evaluated two alternatives: (1) GG 3−5 as an isolated endpoint, and (2) expanding the definition of adverse pathology to include extraprostatic extension (i.e., GG 3−5, ≥pT3a, or LNI (AP-II)). Further, we explored the possibility for Decipher to substratify AP risk in the combined dataset of VL/LR and F-IR patients. Descriptive statistics were provided with medians and ranges reported for continuous variables and frequencies and proportions for categorical variables. Previously determined cut points were used where required for Decipher and CAPRA: (a) Decipher, 0.45 and 0.60 categorized the scores into low-, intermediate- and high-risk groups, respectively [17]; (b) CAPRA scores 0−2, 3−5, 6−10 were grouped as low, intermediate, and high, respectively [16]. In the NCCN + Decipher model, NCCN VL/LR served as the reference group and NCCN F-IR was categorized into Decipher low, intermediate or high.

The performance of each risk assessment tool (e.g., Decipher, CAPRA) was evaluated using multivariable penalized logistic regression modeling with Firth’s method (to account for small event size) [18]. Area under the receiver operating characteristic curves (AUCs) were generated for each model. AUC and its bootstrapped 95% confidence interval were constructed by 1000 resamples and optimism adjustment was applied to multivariable models [19]. Sensitivity analysis of the primary result was performed adjusting institution as a covariate.

All statistical tests were two-sided with *p* values less than 0.05 being considered as statistically significant. No sample size determination was performed prior to retrieving eligible patients from GRID. Analyses were performed in R, version 3.3.1 (R Foundation for Statistical Computing, Vienna, Austria).

## Results

### Patient characteristics in the NCCN F-IR cohort

**Fig. 1:**
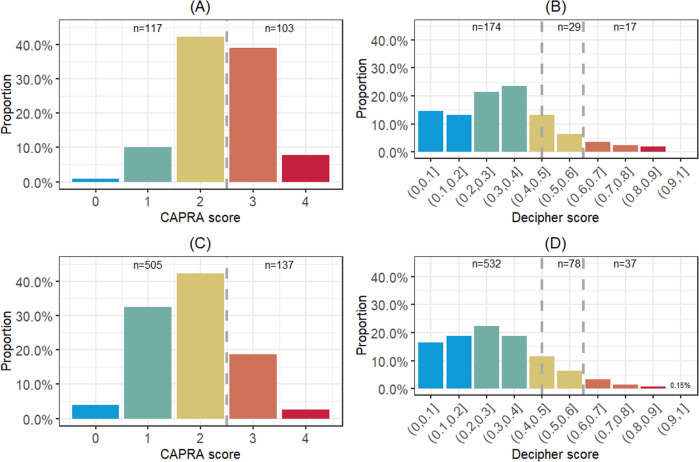
Distribution of **a** CAPRA and **b** Decipher in the F-IR cohort (*n* = 220); **c** CAPRA and **d** Decipher in the combined cohort of VL/LR and F-IR (*n* = 647). CAPRA Cancer of the Prostate Risk Assessment, VL/LR NCCN very low/low risk, F-IR NCCN favorable-intermediate risk

Decipher Biopsy was examined initially for its ability to predict AP post-RP in 220 men with NCCN F-IR disease (Table 1). The median age and PSA at diagnosis was 61 years (interquartile range (IQR) 56–66) and 5.9 ng/mL (IQR 4.6–9.3), respectively. Two-thirds of patients were diagnosed with cT1 and 62% had biopsy GG2 disease. All but two men (who underwent targeted biopsy only) received at least a standard 10 core template biopsy. The CAPRA presurgical clinical risk model classified 53% as low-risk (CAPRA 0−2) and 47% as intermediate-risk (CAPRA 3−4) (Fig. 1a); a large majority of the F-IR patients were CAPRA 2 or 3. The Decipher GC risk model classified 79% (GC < 0.45), 13% (GC 0.45-0.6) and 8% (GC > 0.6) as low-, intermediate- and high-risk, respectively (Fig. 1b). The median time from biopsy to RP was 3 months (IQR 2.4–3). After surgery, 74% had pathological stage pT2, 24% pT3a, 3% pT3b and 18% had positive surgical margins. Lymph node involvement was not observed in any patient. Upgrading from biopsy GG1 to any RP higher GG was observed in 61%, of which only 14% were upgraded to GG3 or greater. Similarly, GG2 upgrading to GG3 or greater was observed in 15% of tumors. Overall 15% had AP at RP, 15% GG 3−5 only and 33% AP-II. Rate of biochemical recurrence at 3 years was 4% with a median follow-up of 2.8 years (IQR: 1.3–5.7).

**Table 1:** Patient characteristics of F-IR cohort (*n* = 220) and VL/LR and F-IR cohort (*n* = 647)

Variables	F-IR only	VL/LR and F-IR
No. patients, *n* (%)	220 (25)	647 (75)
Age		
Median (Q1, Q3)	61 (56−66)	60 (55−65)
Race, *n* (%)		
African American	11 (5)	27 (4)
Caucasian	138 (63)	383 (59)
Other	20 (9)	64 (10)
Unknown	51 (23)	173 (27)
Biopsy stage, *n* (%)		
cT1	147 (67)	474 (73)
cT2	73 (33)	173 (27)
Biopsy grade group, *n* (%)		
Grade group 1	83 (38)	510 (79)
Grade group 2	137 (62)	137 (21)
% positive biopsy cores		
Median (Q1, Q3)	23.5 (14.3, 33.3)	25 (15.4, 35.7)
PSA		
Median (Q1, Q3)	5.86 (4.6, 9.29)	5.47 (4.22, 7)
NCCN, *n* (%)		
F-IR	220 (100)	220 (34)
VL/LR		427 (66)
CAPRA, *n* (%)		
0	2 (1)	25 (4)
1	22 (10)	209 (32)
2	93 (42)	271 (42)
3	86 (39)	120 (19)
4	17 (8)	17 (3)
Unavailable		5 (1)
Time from biopsy to RP (month)		
Median (Q1, Q3)	3 (2.37, 3)	3 (2.73, 3)
RP stage, *n* (%)		
pT2c or less	162 (74)	492 (76)
pT3a	52 (24)	141 (22)
pT3b	6 (3)	14 (2)
RP grade group, *n* (%)		
Grade group 1	56 (25)	273 (42)
Grade group 2	132 (60)	309 (48)
Grade group 3	27 (12)	52 (8)
Grade group 4	4 (2)	9 (1)
Grade group 5	1 (<1)	4 (1)
Positive surgical margins, *n* (%)		
Absent	180 (82)	531 (82)
Present	40 (18)	116 (18)
Lymph node invasion, *n* (%)		
Absent	220 (100)	644 (99.5)
Present		3 (0.5)
BCR follow-up (year) for censored patients		
Median (Q1, Q3)	2.79 (1.33, 5.72)	3.49 (1.47, 6.8)

*F-IR* NCCN favorable-intermediate risk, *VL/LR* NCCN very low/low risk, *PSA* prostate-specific antigen, *NCCN* National Comprehensive Cancer Network, *CAPRA* Cancer of the Prostate Risk Assessment, *RP* radical prostatectomy, *BCR*biochemical recurrence

### Comparison of Decipher and CAPRA for predicting AP

Biopsy Decipher distribution was significantly higher among men with AP at RP (0.38, IQR 0.25–0.49) as compared to those without (0.30, IQR 0.18−0.40, Wilcoxon rank sum test *p* value = 0.016). In univariable analysis (Table 2), the odds ratio (OR) of continuous CAPRA score was 1.6 per 1 unit increased (95% CI 1.0−2.7) and for continuous Decipher score OR was 1.3 per 0.1 unit increased (95% CI 1.1−1.6). Decipher high-risk, but not intermediate-risk, predicted AP with OR of 4.6 (95% CI 1.6−12.9) compared to low-risk groups. In multivariable analysis, Decipher remained a significant predictor. CAPRA had an AUC of 0.60 (95% CI 0.51–0.70) and adding Decipher increased the AUC to 0.65 (95% CI 0.57–0.71) after optimism adjustment. Similar results were observed for two alternative definitions of AP- GG 3-5 and AP-II (Supp. Table 1). Overall, the effect sizes in predicting AP and GG 3−5 are comparable, CAPRA and Decipher alike (e.g., Decipher Univariable-OR 1.36 for GG 3−5 vs. 1.34 for AP) while the effect sizes in predicting AP-II are smaller than the former two (e.g., Decipher Univariable-OR 1.22) as we would expect from a boarder definition of adverse pathology. The sensitivity analysis results in Supplementary Table 2 demonstrated that our observations were robust to institutional effect like local treatment preferences or practices.

**Table 2:** Firth’s penalized logistic regression for Decipher and CAPRA for AP in F-IR cohort (*n* = 220)

Model	Variable	Odds ratio (95% CI)	*p* value	AUC (95% CI)
Univariable	CAPRA	1.64 (1.03−2.68)	0.038*	0.60 (0.51−0.70)
	Decipher	1.34 (1.11−1.63)	0.002*	0.63 (0.52−0.74)
	Decipher int vs. low	0.85 (0.22−2.54)	0.789	—
	Decipher high vs. low	4.60 (1.59−12.90)	0.006*	—
CAPRA + Decipher	CAPRA	1.46 (0.91−2.39)	0.117	0.65 (0.57−0.71)^a^
	Decipher	1.31 (1.08−1.60)	0.006*	
CAPRA + Decipher (risk group)	CAPRA	1.78 (1.10−2.97)	0.018*	—
	Decipher int vs. low	0.61 (0.15−1.91)	0.422	
	Decipher high vs. low	4.92 (1.65−14.41)	0.005*	

Odds ratios of Decipher were reported per 0.1 unit increased

*CAPRA* Cancer of the Prostate Risk Assessment, *AP* adverse pathology, *F-IR* NCCN favorable-intermediate risk

^a^AUC was adjusted for optimism

*p value < 0.05

### NCCN F-IR with Decipher low and intermediate risk groups have same odds of AP as NCCN VL/LR tumors

**Fig. 2:**
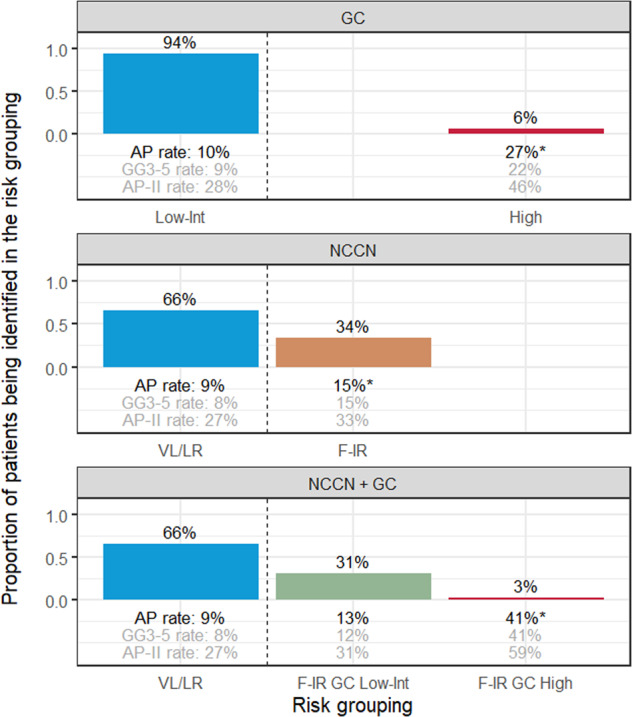
Proportion of combined cohort (*n* = 647) for each risk model and AP rates. Bar heights represent proportions of patients being identified by each risk grouping within a risk model; all three adverse pathology rates are presented under each risk grouping; risk groupings that are significantly associated with AP are indicated by asterisks followed by the AP rates (defined using *p* value < 0.05 from univariable logistic regression models with reference groups either GC Low/Int or NCCN VL/LR). GC Decipher, VL/LR NCCN very low/low risk, F-IR NCCN favorable-intermediate risk

We next analyzed the 220 F-IR patients in comparison to 427 VL/LR tumors (Table 1, Fig. 1c, d). AP rate at RP in the VL/LR tumors (*n* = 427) was 9% and was 11% overall (*n* = 647). Overall, we found NCCN F-IR had increased odds (OR 1.7, 95% CI 1.0−2.8, *p* < 0.05) of AP as compared to NCCN VL/LR tumors. When stratified by genomic risk, only NCCN F-IR with a Decipher high-risk score (but not those with low/intermediate) had a significantly higher odds for AP of 6.8 (*p* < 0.001) compared to VL/LR tumors (Table 3). Decipher was consistently able to stratify further NCCN F-IR tumors for the alternative AP endpoints as well (Supplementary Table 3). As an example, for the AP-II endpoint (which included any pT3 disease at RP) while NCCN F-IR had an OR of 1.3 (95% CI 0.9−1.9, *p* = 0.1), NCCN F-IR with a Decipher high-risk score had an OR of 3.8 (95% CI 1.5−10.4, *p* = 0.006) (Supplementary Table 3b). A bar graph illustrates the risk distributions of the study cohort identified by three risk models (NCCN, GC, and NCCN + GC combined). Corresponding AP rates are annotated below each bar (Fig. 2). Rates of AP in the combined model were low except for the small subset of F-IR tumors with a high Decipher (GC > 0.6) score.

**Table 3:** Firth’s penalized logistic regression for stratification of F-IR (*n* = 220) by Decipher compared to VL/LR (*n* = 427) predicting AP

Model	Variable	Odds ratio (95% CI)	*p* value
NCCN	NCCN F-IR vs. VL/LR	1.71 (1.04−2.79)	0.034*
NCCN stratified by Decipher	NCCN F-IR + GC low vs. NCCN VL/LR	1.48 (0.85−2.53)	0.160
	NCCN F-IR + GC int vs. NCCN VL/LR	1.26 (0.33−3.59)	0.700
	NCCN F-IR + GC high vs. NCCN VL/LR	6.83 (2.45−18.31)	<0.001*

*F-IR* NCCN favorable-intermediate risk, *VL/LR* NCCN very low/low risk, *AP* adverse pathology, *NCCN* National Comprehensive Cancer Network, *GC* genomic classifier–Decipher

*p value < 0.05

## Discussion

The concept of classifying PCa patients by risk group is now 20 years old [20], and while tremendous progress has been made in the intervening decades in our understanding of PCa’s heterogeneous nature and subsequent, complicated prognosis, risk groups are still endorsed by the NCCN and other organizations [21]. Risk groups overweight the importance of T-stage, inconsistently consider the extent of biopsy involvement, do not consider multiple adverse parameters, and do not perform as linear predictors [22]. The addition of the “very low-risk” group and division of intermediate into “favorable” and “unfavorable” has increased granularity, but at the cost of much more complexity, and has not solved any of these fundamental problems. Superior risk prediction using continuous, linear, multivariable tools have been available nearly as long as risk groups [23], and some have been extensively validated [24]. The prognostic value of such tools can be further improved by the incorporation of genomic classifiers.

The NCCN prostate cancer guidelines now subdivide men with intermediate-risk prostate cancer into favorable and unfavorable intermediate-risk groups [6]. Recent studies have shown that men with unfavorable intermediate- and high-risk features demonstrate similar mortality outcomes [9]. Conversely, patients with F-IR tumors demonstrate favorable survival rates and thus may qualify for AS according to NCCN guidelines [8, 9]. However, substantial heterogeneity still prevails within the F-IR category. For example, CAPRA scores in the present F-IR cohort vary from 0 to 4; most had scores of either 2 or 3—and slight majority, mostly those with low—would be classified as “low-risk” by CAPRA. In this study, we demonstrated that among the F-IR tumors, only Decipher high-risk F-IR tumors had increased odds of harboring AP. Moreover, these findings were robust to the definition of AP used.

Appropriately selected AS patients have excellent long-term prostate cancer-specific survival rates [25]. Therefore, some institutions have expanded the inclusion criteria for AS to carefully selected “intermediate-risk” prostate cancer patients to avoid potential overtreatment. However, observational data have shown that these men clearly are at higher risk for upgrading, adverse pathology and progression to metastatic disease [8, 26, 27]—findings that we partly confirmed in our analysis. Patel et al. [10]. aimed in their comparative cohort study to subtype the risk of patients with intermediate-risk prostate cancer for AS based on adverse pathology at radical prostatectomy. They found a threefold greater rate of adverse pathology among men diagnosed with F-IR prostate cancer who underwent radical prostatectomy relative to men at low-risk regardless of the definition used [10]. Notably, the F-IR group also experienced worse survival rates compared to the men with low-risk [10]. Given these results, the authors questioned if we still should offer AS to intermediate-risk patients, even if they show F-IR features.

For patients diagnosed with PCa and variable clinical risk features, genomics classifiers may help overcome some of the limitations of prostate biopsy and disease heterogeneity. One of the advantages of molecular profiling is to assign risk based on an objective estimate of tumor biology, independent of the skills of an individual pathologist [28]. Decipher is a validated genomic classifier based on RNA biomarkers related to cell proliferation and differentiation, motility, immune modulation, and androgen receptor signaling. Recent studies have demonstrated substantial heterogeneity among histologically homogeneous VL/LR and F-IR tumors [13, 28], and have shown that a small subset of patients with GG1 tumors harbor biologically more aggressive disease that may be more appropriately managed by immediate definitive treatment. Interestingly, these studies have also demonstrated that significant proportions of “intermediate-risk” tumors have favorable *molecular* profiles, comparable to low-risk GG1 tumors, suggesting that men with these “indolent” gene expression profiles may qualify for AS [28].

We found that NCCN F-IR had increased odds (1.7 OR) of adverse pathology as compared to NCCN VL/LR tumors, which is consistent with previously reported data [10]. Importantly, however, in these same patients, the Decipher score was a significant predictor of adverse pathology. When stratified by Decipher, we further showed that patients with F-IR cancer with either a Decipher low or intermediate risk group score did not have significantly higher odds of AP. Rather, only the small subset (3% in this study) of F-IR patients with Decipher high-risk results had increased risk of AP (OR of 6.8). Our data suggest that integrating genomic classifiers into treatment decision-making may help identify the most suitable AS candidates among patients with F-IR disease.

Further options that may add value to AS patient selection include the use of multiparametric prostate magnetic resonance imaging, given its high negative predictive value for large high-grade cancers, and novel risk calculators that may increase the number of intermediate-risk patients eligible for AS without increasing the risk of misclassification [29]. However, recent studies have demonstrated that genomic classifiers are more accurate than MRI in predicting the presence of AP [30].

Limitations of this study include its retrospective nature and that certain pathologic features (such as LNI alone, cribriform and intraductal histology, pT3a/pT3b or higher alone) were not explored due to a low event rate. AP is an imperfect endpoint; it is not rare for men to have evidence of AP but to be cured with prostatectomy alone. Due to low event rates and the lack of follow-up data in GRID, oncologic endpoints such as biochemical recurrence, metastasis, and mortality were not evaluated, but these endpoints must be the focus of future studies in the F-IR group. It should be noted that Decipher has already been extensively validated to predict these distal endpoints in higher risk cohorts [31, 32]. In the meantime, AP does continue to drive clinical decision-making, particularly for men with lower clinical risk at diagnosis, and is still the focus of ongoing biomarker studies. Additionally, our patient cohort was derived from multiple centers with varying approaches to workup and follow-up protocols and variable data collection; we do not, for example, have consistent access to PSA density, percent of biopsy tissue involvement, cribriform histology, lymph node count etc. We also do not have long-term PSA serial data post-surgery on any of these men. Despite these limitations, our data emphasize the benefit and potential role of genomic classifiers to better risk-stratify this specific patient population before finalizing treatment decisions.

## Conclusions

The NCCN risk group system has been modified to substratify intermediate risk patients as favorable and unfavorable to address some of its limitations, but these subcategories are still highly heterogeneous clinically and biologically. The Decipher biopsy test can accurately identify patients within the NCCN F-IR group with higher likelihood of AP at the time of RP. Men with NCCN F-IR PCa and a low or intermediate Decipher score had similar odds of AP as men with NCCN VL/LR PCa. Therefore, incorporating Decipher into clinical decision-making, particularly for F-IR PCa, may be useful to safely expand the use of active surveillance.

## Supplementary information

Supp. Table 1

Supp. Table 2

Supp. Table 3
