# Supplementary material for: Decipher identifies men with otherwise clinically favorable-intermediate risk disease who may not be good candidates for active surveillance
Source: Prostate Cancer Prostatic Dis. 2019 Aug 27;23(1):136–43. doi: 10.1038/s41391-019-0167-9 (PMC8076042; doi:10.1038/s41391-019-0167-9)
Supplement: Supplementary file 1 — Supp. Table 1 [file 41391_2019_167_MOESM1_ESM.docx]

Supp. Table 1 - Firth's penalized logistic regression for Decipher and CAPRA for exploratory endpoints in F-IR cohort (n=220): a) GG 3-5; b) AP-II.

a) GG 3-5

| **Model** | **Variable** | **Odds ratio (95% CI)** | **P-value** | **AUC (95% CI)** |
| --- | --- | --- | --- | --- |
| Univariable | CAPRA | 1.60 (1.00 - 2.63) | 0.050* | 0.60 (0.50-0.69) |
|  | Decipher | 1.36 (1.12 - 1.66) | 0.002* | 0.64 (0.53-0.75) |
|  | Decipher Int vs. Low | 0.90 (0.23 - 2.68) | 0.855 | -- |
|  | Decipher High vs. Low | 4.84 (1.67 - 13.61) | 0.005* | -- |
| CAPRA + Decipher | CAPRA | 1.42 (0.88 - 2.34) | 0.148 | 0.65 (0.57-0.71) † |
|  | Decipher | 1.33 (1.10 - 1.63) | 0.004* |  |
| CAPRA + Decipher (risk group) | CAPRA | 1.73 (1.07 - 2.91) | 0.025* | -- |
|  | Decipher Int vs. Low | 0.66 (0.16 - 2.04) | 0.490 |  |
|  | Decipher High vs. Low | 5.15 (1.73 - 15.09) | 0.004* |  |
| *Odds ratios of Decipher were reported per 0.1 unit increased.  † AUC was adjusted for optimism. * P-value < 0.05.* | | | | |

b) AP-II

| **Model** | **Variable** | **Odds ratio (95% CI)** | **P-value** | **AUC (95% CI)** |
| --- | --- | --- | --- | --- |
| Univariable | CAPRA | 1.30 (0.91 - 1.85) | 0.146 | 0.54 (0.47-0.62) |
|  | Decipher | 1.22 (1.05 - 1.42) | 0.010* | 0.60 (0.51-0.68) |
|  | Decipher Int vs. Low | 1.49 (0.65 - 3.30) | 0.337 | -- |
|  | Decipher High vs. Low | 3.36 (1.26 - 9.40) | 0.016* | -- |
| CAPRA + Decipher | CAPRA | 1.20 (0.84 - 1.72) | 0.323 | 0.59 (0.52-0.65) † |
|  | Decipher | 1.20 (1.03 - 1.40) | 0.019* |  |
| CAPRA + Decipher (risk group) | CAPRA | 1.29 (0.90 - 1.88) | 0.161 | -- |
|  | Decipher Int vs. Low | 1.29 (0.55 - 2.92) | 0.553 |  |
|  | Decipher High vs. Low | 3.44 (1.28 - 9.70) | 0.015* |  |
| *Odds ratios of Decipher were reported per 0.1 unit increased.  † AUC was adjusted for optimism. * P-value < 0.05.* | | | | |
