# Supplementary material for: Decipher identifies men with otherwise clinically favorable-intermediate risk disease who may not be good candidates for active surveillance
Source: Prostate Cancer Prostatic Dis. 2019 Aug 27;23(1):136–43. doi: 10.1038/s41391-019-0167-9 (PMC8076042; doi:10.1038/s41391-019-0167-9)
Supplement: Supplementary file 2 — Supp. Table 2 [file 41391_2019_167_MOESM2_ESM.docx]

Supp. Table 2 - Firth's penalized logistic regression for Decipher and CAPRA for endpoints adjusting for site in F-IR cohort (n=220): a) AP; b) GG 3-5; c) AP-II.

a) AP

| **Model** | **Variable** | **Odds ratio (95% CI)** | **P-value** |
| --- | --- | --- | --- |
| Univariable | CAPRA | 1.64 (1.03 - 2.68) | 0.038* |
|  | Decipher | 1.34 (1.11 - 1.63) | 0.002* |
|  | Decipher Int vs. Low | 0.85 (0.22 - 2.54) | 0.789 |
|  | Decipher High vs. Low | 4.60 (1.59 - 12.90) | 0.006* |
| CAPRA + Decipher | CAPRA | 1.23 (0.75 - 2.05) | 0.422 |
|  | Decipher | 1.30 (1.06 - 1.59) | 0.012* |
| CAPRA + Decipher (risk group) | CAPRA Int vs. Low | 1.56 (0.67 - 3.68) | 0.301 |
|  | Decipher Int vs. Low | 0.56 (0.14 - 1.79) | 0.351 |
|  | Decipher High vs. Low | 5.02 (1.62 - 15.42) | 0.006* |
| *Odds ratios of Decipher were reported per 0.1 unit increased.  Institutions are adjusted as a covariate in each model. Institutions with less than 10 samples were grouped. * P-value < 0.05.* | | | |

b) GG 3-5

| **Model** | **Variable** | **Odds ratio (95% CI)** | **P-value** |
| --- | --- | --- | --- |
| Univariable | CAPRA | 1.60 (1.00 - 2.63) | 0.050* |
|  | Decipher | 1.36 (1.12 - 1.66) | 0.002* |
|  | Decipher Int vs. Low | 0.90 (0.23 - 2.68) | 0.855 |
|  | Decipher High vs. Low | 4.84 (1.67 - 13.61) | 0.005* |
| CAPRA + Decipher | CAPRA | 1.19 (0.72 - 2.00) | 0.496 |
|  | Decipher | 1.30 (1.06 - 1.61) | 0.011* |
| CAPRA + Decipher (risk group) | CAPRA Int vs. Low | 1.42 (0.60 - 3.40) | 0.419 |
|  | Decipher Int vs. Low | 0.59 (0.14 - 1.87) | 0.389 |
|  | Decipher High vs. Low | 5.16 (1.66 - 15.88) | 0.005* |
| *Odds ratios of Decipher were reported per 0.1 unit increased.  Institutions are adjusted as a covariate in each model. Institutions with less than 10 samples were grouped. * P-value < 0.05.* | | | |

c) AP-II

| **Model** | **Variable** | **Odds ratio (95% CI)** | **P-value** |
| --- | --- | --- | --- |
| Univariable | CAPRA | 1.30 (0.91 - 1.85) | 0.146 |
|  | Decipher | 1.22 (1.05 - 1.42) | 0.010* |
|  | Decipher Int vs. Low | 1.49 (0.65 - 3.30) | 0.337 |
|  | Decipher High vs. Low | 3.36 (1.26 - 9.40) | 0.016* |
| CAPRA + Decipher | CAPRA | 1.08 (0.74 - 1.59) | 0.675 |
|  | Decipher | 1.20 (1.02 - 1.41) | 0.025* |
| CAPRA + Decipher (risk group) | CAPRA Int vs. Low | 0.85 (0.46 - 1.58) | 0.616 |
|  | Decipher Int vs. Low | 1.38 (0.58 - 3.18) | 0.456 |
|  | Decipher High vs. Low | 3.55 (1.27 - 10.62) | 0.015* |
| *Odds ratios of Decipher were reported per 0.1 unit increased.  Institutions are adjusted as a covariate in each model. Institutions with less than 10 samples were grouped. * P-value < 0.05.* | | | |
