# Supplementary material for: Decipher identifies men with otherwise clinically favorable-intermediate risk disease who may not be good candidates for active surveillance
Source: Prostate Cancer Prostatic Dis. 2019 Aug 27;23(1):136–43. doi: 10.1038/s41391-019-0167-9 (PMC8076042; doi:10.1038/s41391-019-0167-9)
Supplement: Supplementary file 3 — Supp. Table 3 [file 41391_2019_167_MOESM3_ESM.docx]

Supp. Table 3 - Firth's penalized logistic regression for stratification of F-IR (n=220) by Decipher compared to VL/LR (n=427) predicting exploratory endpoints: a) GG 3-5; b) AP-II.

a) GG 3-5

| **Model** | **Variable** | **Odds ratio (95% CI)** | **P-value** |
| --- | --- | --- | --- |
| NCCN | NCCN F-IR vs. VL/LR | 2.03 (1.21 - 3.40) | 0.007* |
| NCCN stratified by Decipher | NCCN F-IR + GC Low vs. NCCN VL/LR | 1.74 (0.98 - 3.04) | 0.060 |
|  | NCCN F-IR + GC Int vs. NCCN VL/LR | 1.56 (0.40 - 4.47) | 0.480 |
|  | NCCN F-IR + GC High vs. NCCN VL/LR | 8.41 (2.99 - 22.77) | <0.001* |
| ** P-value < 0.05.* | | | |

b) AP-II

| **Model** | **Variable** | **Odds ratio (95% CI)** | **P-value** |
| --- | --- | --- | --- |
| NCCN | NCCN F-IR vs. VL/LR | 1.34 (0.94 - 1.90) | 0.109 |
| NCCN stratified by Decipher | NCCN F-IR + GC Low vs. NCCN VL/LR | 1.14 (0.77 - 1.68) | 0.506 |
|  | NCCN F-IR + GC Int vs. NCCN VL/LR | 1.70 (0.77 - 3.62) | 0.183 |
|  | NCCN F-IR + GC High vs. NCCN VL/LR | 3.83 (1.48 - 10.43) | 0.006* |
| ** P-value < 0.05.* | | | |
